# Adverse Childhood Experiences and Forensic Typologies: Getting Specific about Trauma among Institutionalized Youth

**DOI:** 10.3390/ijerph182111307

**Published:** 2021-10-28

**Authors:** Ilma Jahic, Chad R. Trulson, Jonathan W. Caudill, Taea Bonner, Alexandra Slemaker, Matt DeLisi

**Affiliations:** 1 Department of Sociology and Criminal Justice, Iowa State University, Ames, IA 50011, USA; ilmaj@iastate.edu (I.J.); tabonner@iastate.edu (T.B.); slemaker@iastate.edu (A.S.); 2 Department of Criminal Justice, University of North Texas, Denton, TX 76203, USA; chad.trulson@unt.edu; 3 School of Public Affairs, University of Colorado, Colorado Springs, CO 80918, USA; jcaudill@uccs.edu

**Keywords:** adverse childhood experiences, trauma, forensic typologies, crime, violence, psychopathology, juvenile delinquents, developmental psychopathology

## Abstract

Adverse childhood experiences (ACEs) are linked to various conduct and behavior problems within juvenile delinquents, but fewer studies focused on these associations among specific forensic typologies of offending. Utilizing data from 3382 institutionalized delinquents in Texas, logistic regression models indicated multiple associations between ACEs and forensic typologies in both adjusted and unadjusted models, with sexual abuse and physical abuse emerging as the most consistent and robust predictors. Supplemental sensitivity models confirmed the associations between sexual abuse and physical abuse among youth who fit multiple forensic typologies. Models fared poorly at identifying youth who are engaged in fire setting. Implications for total and singular ACEs are discussed, along with how those relate to more clinically meaningful, forensic forms of juvenile delinquency.

## 1. Introduction

A host of developmental factors contributes to conduct problems, delinquency, and serious psychopathology among juveniles. These factors span temperamental, personality, family background, health, peer, socioeconomic, and neighborhood domains [1,2,3,4,5,6]. In recent years, the salience of trauma incurred through adverse childhood experiences (ACEs) has emerged as a dominant research area in criminology [7]. Numerous studies employing data on community, school, clinical, and correctional samples of youth from multiple nations found that greater and more varied trauma exposure was significantly associated with more severe behavioral outcomes [8,9] and greater psychopathology [8,10], and trauma exposure is particularly acute among justice system involved youth [9,11,12,13]. Specifically, adolescent samples from the Fragile Families and Child Wellbeing Study [14], Longitudinal Studies of Child Abuse and Neglect [15], National Survey of Children’s Health [10], and the Project on Human Development in Chicago Neighborhoods [16] reported significant correlations between ACEs and delinquency.

Studies have also shown that cumulative and individual measures of ACEs are associated with specific forms of delinquency including homicide [17], interpersonal aggression [8], robbery and burglary [17], weapons carrying [8], and sexual assault [17,18]. There is also evidence for null or mediated associations between adverse childhood experiences and criminological behavioral outcomes [13,19,20,21]. For instance, Craig et al. [20] studied a sample of 621 serious and violent juvenile offenders and reported extensive trauma histories in their backgrounds. However, ACE score was not associated with re-arrest or violent re-arrest. Instead, juvenile justice history emerged as the strongest predictors of recidivism.

Although researchers provided considerable evidence for the trauma–antisocial behavior relation, there is generally less specificity about the relative effects of specific forms of adverse experiences as well as specific manifestations of conduct problems. Consequently, more recent research examined the associations between adverse childhood experiences among forensic typologies that pertain to specialized manifestations of delinquency or specific forms of psychopathology that are comorbid with conduct problems. Using data from nearly 140,000 students in Minnesota, Duke et al. [8] reported significant associations between trauma exposure and general delinquent involvement, externalizing conduct spanning interpersonal violence and weapons carrying, and internalizing behaviors relating to self-harm, suicidal ideation, and suicide attempts. Among girls and boys, physical abuse, sexual abuse, and three forms of household dysfunction (alcohol use, drug use, and witnessing physical violence) were significantly associated with bullying, physical fighting, dating violence, and weapon carrying. Differential gender effects were found for overall delinquent behavior where physical abuse, sexual abuse, and multiple forms of household dysfunction were associated with reduced delinquency. Among boys, physical abuse, sexual abuse, and household drug use increased delinquent conduct, whereas household alcohol use and witnessing violence were associated with less delinquent conduct.

In a large-scale study using data from 64,000 adjudicated delinquents in Florida, Baglivio et al. [22] found that cumulative ACE exposure was associated with distinct trajectories of delinquent offending, especially more severe variants characterized by earlier onset and greater magnitude of offending. Similarly, Fox et al. [9] found that each additional trauma experience increases the odds of serious, violent, and chronic delinquency by 35%. Among specific adverse childhood experiences, the strongest determinants of serious, violent, and chronic delinquency were incarcerated household member, physical abuse, emotional abuse, physical neglect, household violence, household substance use, and sexual abuse. Emotional neglect and household mental illness had no association with the most pathological delinquent conduct. Perez et al. [23] reported positive correlations between ACE score and substance abuse, mental illness, and serious, violent, and chronic delinquency. Other studies also linked cumulative measures of ACEs to substance use [16], internalizing and externalizing psychopathology [24], and homicidal ideation, planning, attempts, and/or perpetration [25,26].

Against the backdrop of broad associations between assorted trauma exposure, delinquency, and subsequent juvenile justice system contact [7,27,28], there is emerging evidence that adverse childhood experiences also increase liability for more forensic-oriented typologies of offending [9,22,25,26,29]. However, aside from sexual offending, where there is ample research documenting that childhood sexual abuse contributes to subsequent sexual aggression, much less is known about the translation of trauma events into conduct where the youth poses a danger threat to themselves, others, sets fires, or engages in substance use [27]. Given these research gaps, the current study sought to add specificity to the adverse childhood experiences literature by examining their cumulative and individual associations with five forensic typologies with and without adjustments for important delinquent career and demographic covariates.

## 2. Materials and Methods

### 2.1. Participants

Participants are 3382 delinquents adjudicated under a blended sentencing statute and committed to state correctional facilities in Texas between 1987 and 2011. The statute focused on young offenders involved in serious and violent offenses encompassing homicide, kidnapping, sexual assault, felony indecency with a child, robbery, aggravated assault, arson, aggravated or first-degree controlled substance felony, and habitual felony conduct. Youth were placed in juvenile facilities that could extend into adulthood and result in subsequent adult prison placement.

### 2.2. Measures

#### 2.2.1. Adverse Childhood Experiences

Seven dichotomous variables indicating lifetime exposure to adverse childhood experiences were used: sexual abuse (contact sexual offending, prevalence 14%), emotional abuse (caregiver use of intentional infliction of intimidation, coldness, or distress that is verbal, prevalence 18.5%), abandonment (caregiver leaving the child along at home for days or weeks, prevalence 11.5%), medical neglect (caregiver denial of adequate medical care for the youth, prevalence 4.4%), supervision neglect (caregiver is unaware of the child’s location and thus unable to monitor their behavior, prevalence 23%), physical neglect (caregiver refusal to provide shelter, food, or clothing, prevalence 9.2%), and physical abuse (caregiver use of aggression and physical violence toward the child beyond corporal punishment, prevalence 16%). An ordinal measure of family poverty (0 = none, 41% prevalence, 1 = somewhat, 43% prevalence, and 2 = very much, 6% prevalence) indicated that the family had inadequate material resources for daily living. A composite index of total traumatic experiences (M = 1.70, SD = 1.83, range = 0–8) was also used.

#### 2.2.2. Covariates

Prior placements is a count variable (M = 3.69, SD = 4.81, range = −45) which indicated the total out of home placements in the youth’s record prior to current commitment. Prior adjudications is a count variable (M = 2.37, SD = 2.30, range = 0–22) which indicated all court adjudications in the youth’s record prior to current commitment. Age at admission (M = 15.88, SD = 1.17, range = 10.8–18.9) was a continuous variable. Dichotomous terms included white (20.3%), African American (38.9%), and sex (94.5% male, 5.5% female)

#### 2.2.3. Forensic Typologies

Five dichotomous variables (0 = no, 1 = yes) indicated whether the youth was sexually deviant (prevalence = 18.5%), danger to self (prevalence = 18.1%), danger to others (prevalence = 70.3%), fire setters (prevalence = 11.7%), or had a substance abuse problem (prevalence = 50.4%). Typologies were based on behavioral evidence in the youth’s files indicating each specific form of antisocial conduct and were not mutually exclusive. More than 13% of youth fit no forensic typology. Nearly 34% fit one forensic typology and 31% fit two forensic typologies. Nearly 16% of youth fit three typologies, 4.5% fit four typologies, and 1.6% fit all five typologies.

### 2.3. Procedures

The Texas Youth Commission (TYC, since renamed the Texas Juvenile Justice Department) provided de-identified data on youthful offenders across a variety of pre-incarceration domains including information on youth demographics, delinquent histories, family characteristics, adverse childhood experiences, and general measures indicative of antisocial characteristics and other risks. Broadly, these measures derived from a combination of official records, clinical observations of TYC counselors and correctional staff, and on-site diagnostic examinations that occurred at intake to state juvenile correctional facilities. The University of North Texas IRB (application #11321) granted institutional and ethnical approval for the study. As we used archival data, IRB assigned exempt status and written informed consent/assent from the participants was not required to participate in this study in accordance with the national legislation and institutional requirements.

### 2.4. Data Analysis

We employed binary logistic regression with odds ratios for all models in Stata 14.2. Post-estimation tools indicated the percentage of cases that the model correctly classified and the Bayesian Information Criterion (BIC) to allow for comparison across models.

## 3. Results

### 3.1. Logistic Regression Models for Forensic Typologies for Total ACEs without Covariates

Table 1 shows logistic regression models for forensic typologies for total ACEs without covariates. Total ACEs was significantly associated with sexually deviant (OR = 1.33, SE = 0.03, z = 13.35), danger to self (OR = 1.14, SE = 0.03, z = 6.01), danger to others (OR = 1.07, SE = 0.02, z = 3.32), and fire setter (OR = 1.07, SE = 0.03, z = 2.41), but did not have a significant association with substance user. Specifically, each additional adverse childhood experience increased the odds of sexual deviance by 33%, of danger to self by 14%, of danger to others by 7%, and of fire setter by 7%. The percentage of cases correctly classified ranged from 70.31% for danger to others to 88.26% for fire setter.

### 3.2. Logistic Regression Models for Forensic Typologies for Total ACEs with Covariates

Table 2 shows logistic regression models for forensic typologies for total ACEs with covariates. Total ACEs remained significantly associated with sexual deviance, although the effect attenuated slightly to 30% increased odds (OR = 1.30, SE = 0.03, z = 11.89). Females were less likely to be sexually deviant (OR = 0.70, SE = 0.13, z = −1.97). Black delinquents were less likely to be sexually deviant (OR = 0.78, SE = 0.09, z = −2.18), whereas white delinquents were more likely (OR = 2.11, SE = 0.24, z = 6.55). Total ACEs remained significantly associated with danger to self, although the effect attenuated slightly to 9% increased odds (OR = 1.09, SE = 0.03, z = 3.91). Females (OR = 0.60, SE = 0.10, z = −2.95), those younger at admission (OR = 0.87, SE = 0.03, z = −3.72), whites (OR = 2.53, SE = 0.29, z = 8.01), and those with more prior placements (OR = 0.105, SE = 0.01, z = 3.30) were more likely to be dangerous to self.

Total ACEs remained significantly associated with danger to others, although the effect attenuated slightly to 5% increased odds (OR = 1.05, SE = 0.02, z = 2.44). Youth who were younger age admission (OR = 0.88, SE = 0.03, z = −3.82), blacks (OR = 1.31, SE = 0.11, z = 3.24), and whites (OR = 1.43, SE = 0.15, z = 3.38) were more likely to be dangerous to others. With the specification of covariates, total ACEs had no association with fire setter or substance user. Age at admission was inversely associated with fire setter but positively associated with substance user, blacks were less likely to be substance users, and whites were more likely to be fire setters, but less likely to be substance users. Prior placements was positively associated with substance user.

### 3.3. Logistic Regression Models for Forensic Typologies for Individual ACEs without Covariates

Table 3 shows logistic regression models for forensic typologies for individual ACEs without covariates. The effects of specific forms of adverse childhood experiences were highly variable across forensic typologies. Sexual abuse had a large association—532% increased odds—with sexual deviance (OR = 6.32, SE = 0.75, z = 15.54), positive associations with danger to self (OR = 1.80, SE = 0.23, z = 4.67) and danger to others (OR = 1.50, SE = 0.19, z = 3.10), and a negative association with substance use (OR = 0.43, SE = 0.05, z = −7.14). Medical neglect was negatively associated with danger to others (OR = 0.58, SE = 0.13, z = −2.46). Supervision neglect was positively associated with substance user (OR = 1.48, SE = 0.19, z = 3.03). Physical abuse had positive associations with sexual deviance (OR = 1.47, SE = 0.21, z = 2.65) and danger to others (OR = 1.37, SE = 0.19, z = 2.29). Family poverty had positive associations with sexual deviance (OR = 1.19, SE = 0.08, z = 2.57) and substance user (OR = 1.22, SE = 0.08, z = 3.28). Emotional abuse, abandonment, and physical neglect had non-significant associations with all forensic typologies.

Table 4 shows logistic regression models for forensic typologies for individual ACEs with covariates. Once again, the effects of specific forms of adverse childhood experiences were highly variable across forensic typologies. Sexual abuse had significant associations with sexual deviance (OR = 5.54, SE = 0.68, z = 13.95), danger to self (OR = 1.44, SE = 0.19, z = 2.77), and danger to others (OR = 1.46, SE = 0.19, z = 2.82), but no association with fire setter or substance user. Emotional abuse was only associated with substance user (OR = 1.55, SE = 0.20, z = 3.43). Abandonment had null associations with all forensic typologies. Supervision neglect (OR = 1.73, SE = 0.18, z = 5.18) and physical neglect (OR = 1.81, SE = 0.31, z = 3.41) were singularly positively associated with substance user. Physical abuse was positively associated with sexual deviance (OR = 1.38, SE = 0.20, z = 2.19), danger to others (OR = 1.34, SE = 0.19, z = 2.13), and substance user (OR = 1.50, SE = 0.19, z = 3.19). Demographic and delinquent career variables had differential associations. Females were more likely to be a danger to self (OR = 0.63, SE = 0.11, z = −2.62), and youth who were younger at admission were more likely to be a danger to self, danger to others, and fire setters. Blacks were more likely to be a danger to others (OR = 1.32, SE = 0.11, z = 3.28), but had no association with other forensic typologies. In contrast, whites were more likely to be sexually deviant (OR = 1.66, SE = 0.21, z = 4.06), a danger to self (OR = 2.30, SE = 0.28, z = 6.93), danger to others (OR = 1.29, SE = 0.14, z = 2.32), and fire setters (OR = 2.28, SE = 0.32, z = 5.87). Youth with more prior placements were at elevated risk for danger to self (OR = 1.05, SE = 0.01, z = 3.38) and substance user (OR = 1.05, SE = 0.01, z = 4.24).

### 3.4. Supplemental Sensitivity Analyses

Given that many youths fit multiple typologies, we conducted additional models to examine the sensitivity of the main effects. In these Poisson regression models, a count measure for total forensic typologies (M = 1.69, SD = 1.12, range = 0–5) was regressed on the full models with covariates. In model 1, total ACEs was significantly associated with total forensic typologies (IRR = 1.08, SE = 0.01, z = 11.34, *p* < *0*.001). Younger wards, whites, and those with more prior placements also fit more forensic categories. In model 2, individual adverse childhood experiences were specified instead of the summary ACEs measure. Sexual abuse (IRR = 1.23, SE = 0.05, z = 5.38, *p* < *0*.001), physical abuse (IRR = 1.12, SE = 0.05, z = 2.74, *p* < *0*.01), and family poverty (IRR = 1.07, SE = 0.02, z = 3.62, *p* < 0.001) were significantly associated with greater forensic offending. Younger wards, whites, and those with more prior placements also fit more forensic typologies.

## 4. Discussion

Adverse childhood experiences are an emerging research paradigm in criminology and criminal justice [7,11,12,27,28], but research findings often have a mechanistic dose–response connotation and generally lack specificity. This is unfortunate, as although there is universal acknowledgement that childhood abuse and neglect are destructive and contribute to antisocial development and psychiatric morbidity, granular research findings can inform interventions to treat and supervise adolescents involved with the justice system. Several findings bear on measurement issues surrounding adverse childhood experiences, the relative effects of specific forms of trauma, and developmental sequela for violent juveniles with more challenging psychopathology.

Keeping with the title of our study, sexual abuse, physical abuse, or a combination of these two adversities manifest in every forensic typology with the exception of fire setter. Moreover, sexual abuse and physical abuse are robustly associated with meeting criteria for multiple forensic typologies. A unique developmental history is seen for substance users spanning emotional abuse, supervision neglect, physical abuse, and family poverty. Although all forms of abuse and neglect are both preventable and nefarious, not all of them necessarily have significant associations with various delinquent typologies. Physical neglect and abandonment had null effects in all models adjusted with covariates, and emotional abuse, medical neglect, and supervision neglect had significant associations for only one form of forensic offending.

There is value in specifying cumulative and singular measures of adverse childhood experiences as multiple significant effects emerged. Measured cumulatively, total ACE exposure increased the odds of sexual deviance, danger to self, and danger to others with and without covariates. Substantively, these effects were most pronounced for sexual deviance where each additional adverse experience increased the odds by 30% to 33%. Given the overlapping nature of adverse experiences [29,30,31,32,33], a cumulative or summary measure is defensible when examining subsequent effects on offending.

However, we also caution that additive measures of ACEs potentially aggregate disparate forms of adversity that are not at all commensurate in terms of their extremity or behavioral risk. For example, divorce or parental separation is among the most common forms of adversity, with about 80% of justice system involved youth experiencing it. In contrast, about 12% of justice system involved youth were sexually abused [27]. In this regard, parental separation is normative, whereas sexual abuse is pathological in statistical terms, and in terms of the broader psychological and behavioral morbidity it sets into motion [34,35,36,37,38], sexual abuse is incomparable as a form of adversity. Similar criticism is levied toward family poverty, which is commonplace among detained and confined youth. For instance, in Table 4, sexual abuse increases the odds of sexual deviance by 454%, whereas family poverty increases the odds just 26%. Thus, some forms of adverse childhood experiences more accurately reflect adversity, whereas others reflect bona fide trauma.

To borrow a concept from genetics, adverse childhood experiences exhibit pleiotropic effects, that is, specific forms of abuse or neglect manifest in different forensic typologies. This is consistent with research indicating that ACE effects are generally versatile as opposed to being specific to offense type or typology [8,17,18,30,39]. In the current models, this is most evident for sexual abuse where positive associations occur for sexual deviance, danger to self, danger to others, and fire setter, and negative association for substance user. Physical abuse and family poverty also showed versatile effects, whereas other adversities, such as abandonment, had no association with forensic typologies. Disparate forms of abuse and neglect produce differential offending risks.

We acknowledge limitations to the current study primarily centering on omitted variables. Many factors beyond childhood abuse and neglect contribute to the forensic outcomes herein including paraphilic disorders, behavioral disorders, homicidal ideation, psychotic disorders, and diagnostic history [40,41,42,43,44,45,46,47,48,49]. The many null effects for fire setter, for instance, likely occurred as we lacked these variables. This is an important future research stream, namely one that contains psychiatric measures of behavioral disorders, such as Oppositional Defiant Disorder, Conduct Disorder, and Combined Type ADHD, and disparate forms of trauma to see how these conditions independently and interactively result in diverse offense behaviors, such as sexual offending or fire setting. It is likely that youth with more severe externalizing psychopathology and extensive adverse childhood experiences are at greatest risk for the most pathological manifestations of crime [25,26,29].

The ACE indicators were also mostly lifetime binary indicators, which are prone to criticism on methodological grounds for not being able to capture the full extent of childhood trauma in terms of its severity, chronicity, frequency, and source [50,51,52,53]. Our data contain serious delinquent youth who have received deep end juvenile justice placements. As such, the current findings are limited in their generalizability to other adjudicated and justice system involved adolescents, not youth in the general population who have generally less severe trauma histories and certainly less severe offending histories [11,12,22,54].

## 5. Conclusions

ACEs are salient among youth involved in the justice system and within detention centers. The current study examined how adverse childhood experiences were associated with the five typologies (i.e., substance user, sexual deviant, danger to others, fire setter, and danger to self) when adjustments were and were not included for career delinquency and demographic covariates. Specific ACEs manifested in the various typologies. With and without covariates, sexual deviance, along with danger to self and others, had increased odds due to total ACE exposure. Within the detained youth sample, divorced parents and family poverty were common forms of childhood adversity. These findings contribute to the understanding of how traumatic childhood experiences translate into the five typologies.

## Figures and Tables

**Table 1:** Logistic Regression Models for Forensic Typologies for Total ACEs without Covariates.

Variable	Sexually Deviant	Danger Self	Danger Others	Fire Setter	Substance User
	OR (SE)	OR (SE)	OR (SE)	OR (SE)	OR (SE)
	Z	Z	Z	Z	Z
Total ACEs	1.33 (0.03)	1.14 (0.03)	1.07 (0.02)	1.07 (0.03)	0.97 (0.02)
	13.35 ***	6.01 ***	3.32 ***	2.41 *	−1.48
Correctly Classified	81.79%	81.93%	70.31%	88.26%	77.23%
BIC	3077.05	3177.17	4118.54	2547.14	3642.65
Model χ2	176.44 ***	34.37 ***	11.48 ***	5.55 *	2.17

*** *p* < 0.001 and * *p* < 0.05.

**Table 2:** Logistic Regression Models for Forensic Typologies for Total ACEs with Covariates.

Variable	Sexually Deviant	Danger Self	Danger Others	Fire Setter	Substance User
	OR (SE)	OR (SE)	OR (SE)	OR (SE)	OR (SE)
	Z	Z	Z	Z	Z
Total ACEs	1.30 (0.03)	1.09 (0.03)	1.05 (0.02)	1.02 (0.03)	1.00 (0.02)
	11.89 ***	3.91 ***	2.44 *	0.69	0.17
Sex	0.70 (0.13)	0.60 (0.10)	0.99 (0.17)	1.26 (0.32)	0.88 (0.16)
	−1.97 *	−2.95 **	−0.02	0.90	−0.68
Age at Admission	0.97 (0.04)	0.87 (0.03)	0.88 (0.03)	0.77 (0.03)	1.21 (0.04)
	−0.84	−3.72 ***	−3.82 ***	−5.98 ***	5.45 ***
Black	0.78 (0.09)	1.04 (0.11)	1.31 (0.11)	0.98 (0.13)	0.71 (0.07)
	−2.18 *	0.39	3.24 ***	−0.15	−3.42 ***
White	2.11 (0.24)	2.53 (0.29)	1.43 (0.15)	2.28 (0.31)	0.41 (0.05)
	6.55 ***	8.01 ***	3.38 ***	6.08 ***	−8.11 ***
Total Adjud.	0.99 (0.03)	0.99 (0.03)	1.01 (0.03)	1.02 (0.04)	1.01 (0.03)
	−0.10	−0.26	0.23	0.66	0.21
Prior Placements	0.99 (0.02)	1.05 (0.01)	1.01 (0.01)	0.99 (0.02)	1.07 (0.02)
	−0.08	3.30 ***	0.92	−0.10	4.02 ***
Correctly Classified	82.14%	82.05%	70.31%	88.26%	77.47%
BIC	3047.67	3113.11	4132.63	2422.81	3537.19
Model χ2	254.57 ***	147.18 ***	46.14 ***	88.63 ***	156.39 ***

*** *p* < 0.001, ** *p* < 0.01, and * *p* < 0.05.

**Table 3:** Logistic Regression Models for Forensic Typologies for Individual ACEs without Covariates.

Variable	Sexually Deviant	Danger Self	Danger Others	Fire Setter	Substance User
	OR (SE)	OR (SE)	OR (SE)	OR (SE)	OR (SE)
	Z	Z	Z	Z	Z
Sexual Abuse	6.32 (0.75)	1.80 (0.23)	1.50 (0.19)	1.20 (0.19)	0.43 (0.05)
	15.54 ***	4.67 ***	3.10 **	1.13	−7.14 ***
Emotional Abuse	1.08 (0.17)	1.28 (0.19)	1.11 (0.15)	1.21 (0.22)	0.80 (0.12)
	0.48	1.66	0.77	1.05	−1.57
Abandonment	0.88 (0.14)	1.19 (0.18)	0.79 (0.11)	1.27 (0.23)	1.09 (0.17)
	−0.75	1.13	−1.63	1.32	0.58
Medical Neglect	1.18 (0.28)	0.96 (0.22)	0.58 (0.13)	1.07 (0.31)	0.94 (0.22)
	0.70	−0.16	−2.46 *	0.22	−0.29
Supervision Neglect	1.20 (0.16)	0.96 (0.13)	1.10 (0.13)	0.85 (0.14)	1.48 (0.19)
	1.35	−0.29	0.81	−1.01	3.03 **
Physical Neglect	0.89 (0.17)	1.23 (0.22)	1.29 (0.23)	0.82 (0.19)	0.75 (0.13)
	−0.62	1.14	1.43	−0.85	−1.59
Physical Abuse	1.47 (0.21)	1.21 (0.17)	1.37 (0.19)	1.30 (0.22)	1.12 (0.16)
	2.65 **	1.31	2.29 *	1.53	0.8
Family Poverty	1.19 (0.08)	0.89 (0.06)	1.00 (0.06)	1.00 (0.08)	1.22 (0.08)
	2.57 **	−1.74	0.04	0.06	3.28 ***
Correctly Classified	82.76%	81.93%	70.34%	88.26%	77.20%
BIC	2932.82	3199.95	4147.69	2505.99	3619.35
Model χ2	374.55 ***	68.47 ***	39.21 ***	13.58	82.36 ***

*** *p* < 0.001, ** *p* < 0.01, and * *p* < 0.05.

**Table 4:** Logistic Regression Models for Forensic Typologies for Individual ACEs with Covariates.

Variable	Sexually Deviant	Danger Self	Danger Others	Fire Setter	Substance User
	OR (SE)	OR (SE)	OR (SE)	OR (SE)	OR (SE)
	Z	Z	Z	Z	Z
Sexual Abuse	5.54 (0.68)	1.44 (0.19)	1.46 (0.19)	0.93 (0.15)	0.82 (0.10)
	13.95 ***	2.77 **	2.82 **	−0.42	−1.67
Emotional Abuse	1.02 (0.16)	1.15 (0.17)	1.07 (0.15)	1.06 (0.19)	1.55 (0.20)
	0.10	0.91	0.49	0.31	3.43 ***
Abandonment	0.92 (0.15)	1.25 (0.20)	0.78 (0.11)	1.34 (0.25)	1.12 (0.15)
	−0.54	1.46	−1.74	1.59	0.83
Medical Neglect	1.23 (0.29)	1.01 (0.24)	0.59 (0.13)	1.18 (0.35)	1.03 (0.25)
	0.86	0.03	−2.34 *	0.56	0.12
Superv. Neglect	1.23 (0.16)	0.94 (0.13)	1.08 (0.12)	0.86 (0.14)	1.73 (0.18)
	1.51	−0.46	0.68	−0.98	5.18 ***
Physical Neglect	0.85 (0.16)	1.18 (0.21)	1.27 (0.23)	0.78 (0.18)	1.81 (0.31)
	−0.85	0.91	1.33	−1.03	3.41 ***
Physical Abuse	1.38 (0.20)	1.08 (0.16)	1.34 (0.19)	1.14 (0.20)	1.50 (0.19)
	2.19 *	0.53	2.13 *	0.78	3.19 ***
Family Poverty	1.26 (0.09)	0.94 (0.06)	1.00 (0.06)	1.06 (0.08)	1.49 (0.08)
	3.36 ***	−0.96	0.07	0.78	7.41 ***
Sex	0.95 (0.18)	0.63 (0.11)	1.07 (0.18)	1.24 (0.32)	0.94 (0.15)
	−0.29	−2.62 **	0.42	0.85	−0.37
Age at Adm.	0.99 (0.04)	0.87 (0.03)	0.88 (0.03)	0.76 (0.03)	0.96 (0.03)
	−0.33	−3.68 ***	−3.65 ***	−6.07 ***	−1.25
Black	0.80 (0.09)	1.03 (0.11)	1.32 (0.11)	0.97 (0.13)	0.90 (0.07)
	−1.91	0.28	3.28 ***	−0.20	−1.26
White	1.66 (0.21)	2.30 (0.28)	1.29 (0.14)	2.28 (0.32)	1.14 (0.12)
	4.06 ***	6.93 ***	2.32 *	5.87 ***	1.27
Total Adjud.	1.01 (0.03)	0.99 (0.03)	1.01 (0.03)	1.03 (0.04)	0.98 (0.02)
	0.17	−0.19	0.35	0.68	−0.69
Prior Placements	1.00 (0.02)	1.05 (0.01)	1.01 (0.01)	0.99 (0.02)	1.05 (0.01)
	0.09	3.38 **	0.94	−0.10	4.24 ***
Correctly Classified	83.09%	81.87%	70.28%	88.23%	61.56%
BIC	2952.04	3150.71	4157.94	2466.75	4536.49
Model χ2	407.09 ***	158.34 ***	69.59 ***	93.45 ***	265.52 ***

*** *p* < 0.001, ** *p* < 0.01, and * *p* < 0.05.

## Data Availability

Data are not available for distribution.
